# Association between Serum Uric Acid Levels, Nutritional and Antioxidant Status in Patients on Hemodialysis

**DOI:** 10.3390/nu12092600

**Published:** 2020-08-27

**Authors:** Etna Domínguez-Zambrano, José Pedraza-Chaverri, Ana Laura López-Santos, Omar Noel Medina-Campos, Cristino Cruz-Rivera, Francisco Bueno-Hernández, Angeles Espinosa-Cuevas

**Affiliations:** 1 Department of Nephrology and Mineral Metabolism, Instituto Nacional de Ciencias Médicas y Nutricion Salvador Zubirán, México City PC 14080, Mexico; etna.lucero@gmail.com (E.D.-Z.); analaura.9403@gmail.com (A.L.L.-S.); cristino.cruzr@incmnsz.mx (C.C.-R.); 2 Medicine Faculty, Universidad Nacional Autónoma de México, México City ZC 04510, Mexico; 3 Chemistry Faculty, Universidad Nacional Autónoma de México, México City ZC 04510, Mexico; pedraza@unam.mx (J.P.-C.); omarnoelmedina@gmail.com (O.N.M.-C.); 4 Centro de Atención Renal, Estado de México ZC 53100, Mexico; francisco.bueno@hemocare.com.mx

**Keywords:** uric acid, nutritional status, renal dialysis, oxidative stress, malondialdehyde, oxygen radical absorbance capacity, 2,2-diphenyl-1-picrilhydrazyl

## Abstract

Purpose: To determine the relationship between uric acid (UA) and nutritional and antioxidant status in hemodialysis (HD) patients, given that hyperuricemia could be an indicator of good nutritional status possibly because of the antioxidant properties of UA. Methods: Cross-sectional study with 93 patients on HD. Hyperuricemia was considered as UA ≥6.0 mg/dL in females and ≥7.0 mg/dL in males. Nutritional variables were registered. Blood samples were taken before the dialysis session to determine oxidative damage as plasma malondialdehyde (MDA) content, and antioxidant capacity measuring 2,2-diphenyl-piclrylhidrazil radical (DPPH^●^) scavenging activity and oxygen radical absorbance capacity (ORAC) value. Results: Patients with hyperuricemia had higher creatinine (11.9 vs. 10.5 mg/dL; *p* = 0.004), potassium (5.5 vs. 5.0 mg/dL; *p* = 0.014) levels; phase angle (5.8 vs. 4.9; *p* = 0.005), protein consumption (normalized protein nitrogen appearance, nPNA, 1.03 vs. 0.83; *p* = 0.013) than normouricemic patients. DPPH^●^ scavenging activity was higher in hyperuricemic subjects (1.139 vs. 1.049 mM Trolox equivalents; *p* = 0.007); likewise, hyperuricemic subjects had less oxidant damage measured by MDA (10.6 vs. 12.7 nmol/mL; *p* = 0.020). Subjects with normouricemia were at higher risk of having a reactance to height (Xc/H) ratio less than 35 (OR 2.79; 95% CI, 1.1–7.017, *p* = 0.028); nPNA < 1.0 (OR 3.78; 95% CI, 1.4–10.2, *p* = 0.007), diagnosis of cachexia (OR 2.95; 95% CI, 1156–7.518, *p* = 0.021), potassium levels <5 (OR 2.97; 95% CI, 1.136–7.772, *p* = 0.023) and PA < 5.5° (OR 3.38; 95% CI, 1.309–8.749, *p* = 0.012.) Conclusions: Patients with hyperuricemia had higher antioxidant capacity and better nutritional status. Purines and protein restrictions in HD patients with hyperuricemia need to be reviewed individually for each patient. More studies are needed to stablish a cut point of UA levels in renal population.

## 1. Introduction

Chronic kidney disease (CKD) is a public health problem given its epidemic nature, the complications it causes and the lack of human and material resources available for its treatment [1,2,3]. End stage renal disease (ESRD) patients are at high risk of protein energy wasting (PEW) syndrome (a pathological entity characterized by low protein and energetic deposits and systemic inflammation), and their nutritional status should be thoroughly assessed to diagnose and treat PEW in time [4,5]. The diet of renal patients on hemodialysis (HD) is restricted in different nutrients, such as potassium (K), phosphorus (P) and sodium (Na) [6,7], in addition to purines when hyperuricemia is detected [8,9]. These dietary restrictions, together with other dietary factors and HD, may increase the risk and/or aggravate PEW [10]. Uric acid (UA) is the final product of purine metabolism [11]. Hyperuricemia, defined as UA levels ≥6.0 and ≥7.0 mg/dL in women and men, respectively [12], has been related with cardiovascular (CV) events; for example, in the URRAH Project cohort, the level of uricemia above which the independent risk of CV disease may increase are 4.7 and 5.6 mg/dL for total and CV mortality, respectively [13]. Furthermore, hyperuricemia has been associated with the incidence of CKD in healthy subjects [14]; one of the reasons has been UA role as an oxidizing molecule [15]. However, there are controversial data about the latter in the HD population, in whom it also has a role as an antioxidant molecule (due role of UA) [16,17]. Recent studies [18,19,20,21] have pointed to UA as a marker of good nutritional status in subjects on HD, as it has been associated with adequate values of body mass index (BMI), handgrip strength and protein intake. However, these studies had different nutritional objectives and did not evaluate the nutritional status of patients or the presence of PEW with an adequate number of markers, as suggested by international institutions, nor did they attempt to measure oxidant damage to provide evidence of the dual role of UA in this population [22,23,24]. The objective of the present study was to establish an association between UA concentration and nutritional and antioxidant status.

## 2. Materials and Methods

We studied patients attending two HD units in our city. Inclusion criteria were as follows: At least three months on chronic HD; without amputations or implants or comorbidities; and without difficulties in mobility or behaviors that affected UA concentrations. Patients with a history of alcohol or drug use, liver cirrhosis, comorbidities and/or treatments (neoplasms, active autoimmune and infectious diseases or chemotherapy) that affected UA levels were excluded. To calculate sample size, we used statistical tables for odds ratio (OR) estimations according to the data of the Lee et al. study [23].

The institutional ethics committee authorized the protocol with the number 1968. All patients signed an informed consent form. Hyperuricemia was defined as serum UA ≥6.0 mg/dL in women and ≥7.0 mg/dL in men [12,25]. Dialysis efficacy was measured by Kt/V and urea reduction rate (URR) indicators. From the patients′ medical records, we obtained data about the dialysis characteristics and normalized protein nitrogen appearance (nPNA). We also conducted predialysis biochemical measurements of phosphorus, potassium, transferrin, creatinine and blood urea nitrogen (BUN).

### 2.1. Primary Outcomes: Antioxidant Status.

Before the beginning of the HD session, blood samples were taken (with at least 4 h fasting), directly using vascular access to analyze the markers of antioxidant capacity (2,2-diphenyl-piclrylhidrazil radical (DPPH^●^)) scavenging activity (a single electron transfer (SET)-based assay) and oxygen radical absorbance capacity (ORAC) value (a hydrogen atom transfer (HAT)-based assay) as well as the content of oxidative damage marker malondialdehyde (MDA). The samples were collected in tubes with the anticoagulant ethylenediamine tetraacetic acid (EDTA; 7.2 mg), centrifuged for 15 min at 175× *g*, placed in 1.5 mL centrifuge tubes and stored for approximately 10 days at −40 °C before the analysis. The DPPH^●^ scavenging assay was performed according to the colorimetric method described by Koren et al. [26] and results were expressed as mM Trolox equivalents (TE): Fluorometric ORAC assay was performed according to Huang et al. [27] and data were expressed as uM TE. Finally, the spectrophotometric assay of MDA was performed according to the procedure described by Gérard-Monnier et al. [28] and the measurements were expressed as nmol MDA/mL. All determinations were obtained by using Synergy HT multi-mode microplate reader (BioTek Instruments Inc., Winooski, VT, USA).

### 2.2. Secondary Outcomes: Nutritional Status

After HD, nutritionists trained in anthropometric techniques assessed the nutritional status. The BMI was estimated based on the weight and height of each patient (weight scale SECA^®^, Germany). A Lange caliper (Beta Technologies, Santa Cruz, CA, USA) was used to measure skin folds, and a Takei dynamometer was used to measure handgrip strength by asking the patients to squeeze the dynamometer three times using the arm opposite to the vascular access (fistula or catheter) and recording the highest value. To diagnose PEW, we used the malnutrition inflammation score (MIS ≥6 points) and the criteria from the International Society of Renal Nutrition and Metabolism (ISRNM, biochemical criteria; low body weight, reduced total body fat, or weight loss; a decrease in muscle mass; and low protein or energy intakes) [10,29]. To evaluate the diet, the patients were asked to record the food they consumed on three days: One day without hemodialysis, one day with hemodialysis and one day of the weekend (the analysis was performed using the NutriKcal VO software). Bioelectrical impedance was measured using Body-Stat Quadscan 4000 and RJL equipment; we obtained bodily dielectric properties as follows: Resistance normalized per height (R/H), reactance normalized per height (Xc/H) and phase angle (PA). For the diagnosis of cachexia [30], we carried out a bioelectrical impedance vector analysis with the software BIVA-confidence and BIVA-tolerance by Piccoli et al. [31,32], using the reference values of the Mexican population [33].

### 2.3. Statistical Analysis

The data are reported as means ± standard deviations (SD), as medians and interquartile ranges (IQR), or as frequencies and percentages, according to the Kolmogorov–Smirnov and Shapiro–Wilk tests. Student′s *t*-test was used to compare the variables of independent samples. The Mann–Whitney U test and the chi-square test were used as necessary. The bivariate analysis used to estimate risks (with 95% confidence intervals) included a single-step logistic regression. A value of *p* < 0.05 was considered statistically significant. The statistical program SPSS v.21 (IBM Corporation, Chicago, United States of America) was used to analyze the data.

## 3. Results

We studied 93 patients of two HD units; 54% were men, and the median age of all patients was 40 (29–52) years. The prevalence of hyperuricemia was 72% (95% CI: 67–77%) and that of PEW, according to MIS, was 35% (95% CI: 30–40%). In the general population the time of dialysis was 215.3 ± 17.3 min, and the dialysis vintage was 39 (21–79) months; the medium-flux filter (F80) was the most common filter used for the HD session (69%) and dialysis efficacy measured by Kt/V was 1.46 (1.25–1.77) and URR was 71% (65–79). The majority of the patients had a dialysis session three days a week; an arteriovenous fistula (AVF) was the predominant type of vascular access, and the efficacy of hemodialysis, as measured by Kt/V and URR, was adequate in all patients (Table 1).

### 3.1. Primary Outcomes: Antioxidant Status

The total antioxidant capacity, as measured by DPPH^●^ scavenging, was higher in patients with hyperuricemia (1.14 vs. 1.05 EqMm of Trolox), who also showed lower oxidative damage, as measured by MDA (10.6 vs. 12.7 nmol/mL) (Table 2).

### 3.2. Secondary Outcomes

The values of the biochemical parameters measured in the study are shown in Table 3. Patients with hyperuricemia had higher concentrations of creatinine (11.9 vs. 10.5 mg/dL; *p* = 0.004), predialysis BUN (70 vs. 53 mg/dL: *p* < 0.001) and potassium (5.5 vs. 5.0 mg/dL; *p* = 0.014). In general, all patients had adequate albumin and transferrin levels, adequate nutritional status (MIS score) and adequate handgrip strength values.

The anthropometric markers of body composition indicated that subjects had an adequate BMI and a good percentage of fat and lean mass. Regarding the electrical properties of the body, patients with hyperuricemia had a higher ratio of reactance to height (Xc/H) (37.6 vs. 30.2; *p* = 0.004) and PA values (PA, 5.8 vs. 4.9°, *p* = 0.005), as well as a lower prevalence of cachexia (54%; 95% CI = 49–59%). Likewise, the nutritional intake analysis showed that patients with hyperuricemia had a higher protein intake, based on the nPNA values (1.03 vs. 0.83; *p* < 0.05) and the animal protein intake (37.1 vs. 32.1 g/d; *p* = 0.017) (Table 4).

**Figure 1:**
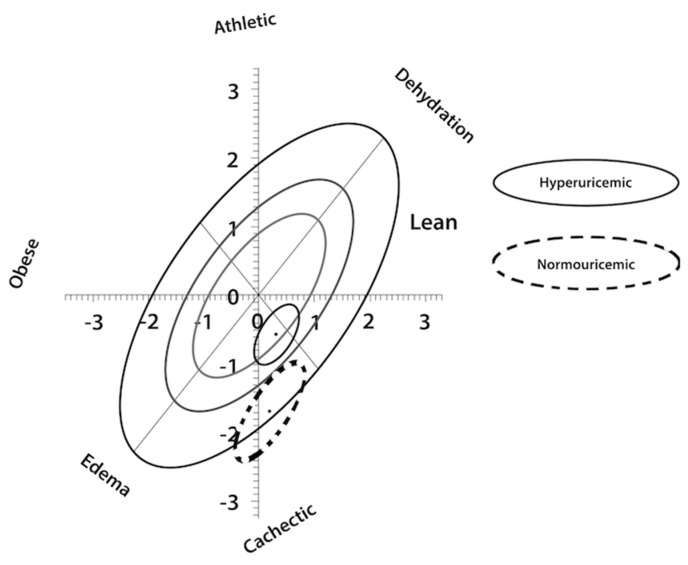
Z Score of normouricemic and hyperuricemic patients by Bioelectrical Impedance Vector Analysis (BIVA). Continuous line represent hyperuricemic subjects and dotted line represent normouricemic subjects.

Regarding body composition and cachexia diagnosis by bioelectrical impedance vector analysis (BIVA), the subjects with hyperuricemia had better nutritional and hydration status than patients with normouricemia and diagnosis of cachexia and volume overload (Figure 1).

To evaluate the association between UA, nutritional status and antioxidant status, we performed logistic regressions analysis to determine which indicator of nutritional status was better explained by hyperuricemia. In the model adjusted for sex and URR, albumin ≥3.5 g/dL was independently associated with UA levels; *p* < 0.05; R2 = 0.541 (90.3% of the subjects). The model included other nutritional variables, such as kilocalories consumed per day, total kilocalories and grams of protein, kilocalories and grams of protein per kilogram of ideal weight, and handgrip strength measurements (Table 5).

A risk analysis was carried out to identify variables that could be indicators of nutritional wasting along with hyperuricemia. The analysis showed that subjects with normouricemia were at higher risk of having a reactance to height (Xc/H) ratio less than 35 (OR 2.79; 95% CI, 1.1–7.017, *p* = 0.028); nPNA < 1.0 (OR 3.78; 95% CI, 1.4–10.2, *p* = 0.007), diagnosis of cachexia (OR 2.95; 95% CI, 1156–7.518, *p* = 0.021), potassium levels <5 (OR 2.97; 95% CI, 1.136–7.772, *p* = 0.023) and PA < 5.5° (OR 3.38; 95% CI, 1.309–8.749, *p* = 0.012 (Table 6).

## 4. Discussion

In numerous aspects, serum UA appears to be a two-faced marker in health and disease [34]. In the early stages of renal disease, UA is a risk factor marker for mortality, whereas during peritoneal dialysis treatment, it is a prognostic factor [35]. However, its role in hemodialysis patients is not very clear; similar than a reverse epidemiology phenomenon (paradoxical relationships between nutritional markers such as increased weight or high serum cholesterol levels that are protective in dialysis patients), could be associated with better satisfactory outcomes [36,37]. Regarding general mortality, in a study of 7333 HD patients, hyperuricemia correlated with lower mortality in the multivariate models analysis. Furthermore, UA concentrations correlated positively with nutritional markers such as BMI, nitrogen protein catabolic rate (nPCR) and phosphorus levels [38]. The latter finding has been attributed to a higher antioxidant capacity in patients with hyperuricemia, since it has been observed that UA protects cell membranes against lipid peroxidation and act like a free radical scavenger [34]. In children, UA has been proposed as a diagnostic tool for CKD progression, since its levels increased as do the antioxidant capacity, measured in saliva by ferric ion reducing antioxidant (FRAP) assay, according to the severity of CKD [39]. However, it is worth mentioning that the interplay between hyperuricemia, oxidative damage and nutritional status has not yet been explored.

To our best knowledge, this is the first study that explores the association between UA, nutritional status and antioxidant capacity and oxidative damage in HD patients. Since antioxidants inactivate free radicals through two mechanisms—SET (single electron transfer) or HAT (hydrogen atom transfer)—the antioxidant capacity was evaluated by the DPPH^●^ scavenging and ORAC assays. ORAC is a HAT-based assay and DPPH radical scavenging is a SET-based assay. We are tempted to speculate that the differences in both assays may explain the fact that only the DPPH^●^ scavenging ability was significant between normouricemic and hyperuricemic patients. Patients with hyperuricemia had higher antioxidant capacity, as measured by DPPH^●^ scavenging activity, and less oxidative damage, as measured by MDA. Uric acid plays a role as an antioxidant or oxidant in different clinical scenarios. Although fruits and vegetables have abundant amounts of dietary antioxidants, they cannot adequately represent the overall ability and interactions of antioxidants in the whole diet. For example, in our study we analyzed the consumption of food groups, and the consumption of fruits and vegetables that could have a greater association with antioxidant capacity was only 1.3 servings of fruits (0.7 to 1.9 in hyperuricemic, and 0.8 to 2.2 in normouricemic) and 2 servings of vegetables (1.3 to 3.4 in hyperuricemic, and 1.2 to 2.4 in normouricemic). Thus, the dietary concept which takes the whole dietary antioxidants into account has become more prominent. Dietary total antioxidant capacity (DTAC) is a novel indicator of diet quality [40], which is used to estimate the cumulative power of antioxidants in the whole diet [41]. Recently, DTAC is described as an effective tool for determining the health outcomes in populations [42,43]. This new approach could serve for future lines of research to understand the behavior and association of uric acid, antioxidant status and diet in these patients [44].

In respect to our secondary outcomes, Beberashvili et al. [18,19] studied geriatric patients to find an association between UA and nutritional status. In contrast, we studied a relatively young population (median age was 40 (29–52) years) and found similar results; in our country, a dialysis registry is lacking but the mean age of people with CKD is 44.8 ± 17.2 years old [45,46]. In our population, the main etiology of CKD was unknown, contrary to that described in the studies by Abbas et al. [47] and Hsu et al. [48], in which the main causes were diabetic nephropathy and arterial hypertension. We found a prevalence of PEW (score MIS ≥6) of 35%, a low prevalence compared to those usually found in HD patients worldwide, which ranges from 50% [49] according to MIS, to 52.5% [50] and 70% [51] according to ISRNM criteria.

One of the main findings of our study was the existence of clinical and statistically significant differences between hyperuricemia and normouricemia, in the variables of nutritional status according to the logistic regression model between UA and albumin, which appropriately classified 90.3% of the study subjects. No independence was found between UA and other nutritional variables, likely because the nutritional status is a construct made up of different variables (the logistic regression data are not shown).

All patients had similar dialytic characteristics, but patients with hyperuricemia had higher creatinine, predialysis BUN and potassium levels. In patients with normouricemia, the risk of having potassium levels lower than 5 mg/dL was 1.97 times higher than in patients with hyperuricemia. Regarding the biochemical variables of nutritional status, patients with normouricemia had higher concentrations of creatinine and potassium. In the study by Beberashvili et al. [18], creatinine levels were higher in the third tertile than in the first (8.43 ± 2.2 vs. 6.51 ± 2 mg/dL); the same authors found that albumin levels were also higher in the last tertile (3.9 ± 0.3 vs. 3.7 ± 0.4, *p* < 0.001), although no significant differences were found in our study.

Some variables (biochemical parameters such as phosphorus, albumin, hemoglobin, transferrin levels and anthropometric parameters such as BMI, arm circumference, triceps skinfold and percentage of body fat) were similar between both study groups, which could explain why no differences were observed in the final score or in the diagnosis of PEW between patients. In the cohort of dialysis patients studied by Bae et al. [22], UA levels <5.5. mg/dL were associated with overall mortality (adjusted HR of 1.720; 95% CI, 1007–2937, *p* = 0.047); there was also a positive correlation between UA levels <5.5 mg/dL and nutritional factors such as BMI (regression coefficient [B] = 0.042; *p* = 0.091), Subjective Global Assessment (SGA) (B = −0.062, *p* = 0.014), serum phosphorus (B = 0.107, *p* < 0.001) and albumin (B = 0.127, *p* < 0.001). Among patients with low levels of UA (<5.5 mg/dL), there was a higher proportion of malnourished subjects, according to SGA score, lower BMI and lower concentrations of total proteins, phosphorus and serum albumin. In the study by Beberashvili et al. [18], mean MIS score was lower in the hyperuricemia group (5.06 ± 3.1 mg/dL) than in the normouricemia group (7.44 ± 3.5 mg/dL) (*p* < 0.001). In contrast, in our study we did not find differences between the scores of the groups classified according to UA levels. Although hyperuricemia was not statistically significant in the logistic regression models used in our study for some markers of nutritional status, including it in the model did not decrease the value of R^2^ or the percentage of patients it was able to accurately classify (the model explained 48.6% of the PEW and accurately classified 80.6% of the population).

In our study, handgrip strength values were very similar between both groups of subjects, which could be associated with the fairly similar prevalence of PEW among the population. Other studies found that low muscle strength, as measured by dynamometry, was associated with PEW and was considered a nutritional marker associated with muscle loss [52]. Some studies have even found a positive correlation (r = 0.26; *p* < 0.001) between handgrip strength and UA levels [18].

In contrast, the analysis of body composition by BIVA revealed differences in dielectric properties; patients with hyperuricemia had higher values of R/H and Xc/H than patients with normouricemia, indicating better body cell mass and lower volume overload. The risk that patients with normouricemia would have a ratio of Xc/H lower than 35 Ω/m (worse body cell mass) was 1.8 times greater than that for patients with hyperuricemia. Few studies have analyzed the BIVA values of patients on HD and their relationship with UA; most have analyzed only total body water, extracellular water, fat mass and fat-free mass values. One of the indicators of BIVA with the highest prognostic value in patients on HD is the PA, which has been demonstrated to have an inverse relationship with mortality (the greater the PA is, the lower the risk of mortality); for example, studying a cohort of 91 patients on HD, Beberashvili et al. [53] reported that for each 1° increase in PA, the crude and adjusted mortality HR were 0.6 (95% CI 0.54–0.71) and 0.61 (95% CI 0.53–0.71), respectively. In the present study, PA values were higher in patients with hyperuricemia than in those with normouricemia.

Furthermore, cachexia (the most severe form of malnutrition in dialysis patients) was lower in patients with hyperuricemia. In our study the risk that patients with normouricemia would have a PA lower than 5.5° was 2.28 times higher than that for patients with hyperuricemia; the risk of having cachexia, diagnosed by bioelectrical impedance vector analysis, according to Piccoli et al. [32] was 1.9 times higher. Figure 1 shows that the ellipse of patients with normouricemia is within the range of cachexia and volume overload, while patients with hyperuricemia had better nutritional and hydration status. There are no studies that use impedance vectors as variables of nutritional and hydration status and analyze their association with UA levels.

In addition to the changes induced by CKD in the metabolism of UA, dietary and pharmacological interventions, as well as the nature and extent of dialysis treatments, greatly modify the concentration of UA in the population with CKD. The intake of kilocalories consumed per day was the same in both groups, both in kilocalories/kg of actual and ideal weight. For proteins, contrary to what the dietary restrictions in the intake of purines for patients with hyperuricemia have led us to expect, patients had a higher protein intake as measured by grams of protein/kg of ideal weight and intake of protein of animal origin.

It has been widely described in the literature that the caloric and protein intake of HD patients is below the recommendations of the KDIGO guidelines (30 to 35 kcals/kg/d and 1.2 g/d of calories and proteins, respectively). The reported average caloric intake has ranged from 23.9 ± 7.01 to 24.8 ± 7.5 kcals/d, while the average protein intake has ranged from 0.98 ± 0.30 to 1.1 ± 0.4 g/d. Luis et al. [6] reported that only 11% and 41% of patients on HD meet the caloric and protein intake requirements, respectively. The nPNA, a more specific indicator of protein intake, was higher in patients with hyperuricemia. Beberashvili et al. [18] obtained similar results: 1.10 ± 0.27 vs. 1.00 ± 0.20 in the third and first tertile of UA of their study population, respectively. In our study, the risk that patients with normouricemia would have a nPNA below 1.0 was 2.8 times higher than for patients with hyperuricemia. In contrast with what is true for the general population, low UA levels (but not high UA) are associated with higher mortality from all causes for HD patients, especially when they have a low protein intake. In fact, protein-rich diets tend to contain large amounts of purines, so that the higher concentrations of UA could indicate a better nutritional status in the population with ESRD [20].

In general, the diet of patients on HD is very low in fruits, vegetables, legumes and dairy products (foods rich in antioxidants in the first two and in proteins in the latter one). In our study, this was true for both groups of patients. Fructose levels, a variable that has been implicated in the increase in UA levels, were not different between groups (data not shown), likely because patients tend to underreport the intake of sugar and high-fructose foods in the food record.

The most important strength of our study is that it includes measurements of antioxidant capacity and oxidative damage. Furthermore, it is possible that a low concentration of UA results in a lower total antioxidant capacity in patients on dialysis, although further research is needed to clarify these mechanisms.

Cohort studies have described a phenomenon of reverse epidemiology on HD patients, where high concentrations of UA are a protective factor against mortality and morbidity. Those studies have separately measured some variables of nutritional status and concluded that high UA levels are an indicator of better nutritional status, as evidenced by the positive association between UA levels and nutritional status variables. Furthermore, low concentrations of UA are considered a consequence of poor protein intake and the presence of malnutrition. Hsu et al. [48] found a positive correlation (r = 0.518, *p* < 0.001) between UA and predialysis BUN (indicator of protein intake), a similar result to that obtained in the present study, where the corresponding values were r = 0.479 and *p* < 0.001. This phenomenon of reverse epidemiology has been used to explain an improved antioxidant capacity of UA in vivo and in vitro, where it has contributed up to 60% of the elimination of free radicals in blood serum.

The results obtained in this study should be supported by measuring other markers of oxidative damage (more specific for proteins and DNA) to achieve a better understanding of the underlying mechanisms that promote antioxidant capacity in subjects with hyperuricemia on HD. This would also support the associations and correlations that have been found between hyperuricemia, some parameters of nutritional status and mortality, which have suggested the existence of a phenomenon of reverse epidemiology in HD patients with respect to the role of UA (similar to creatinine, potassium and BMI values in dialysis patients) [37]. Rethinking UA as a laboratory marker of nutritional status would require changing the dietary guidelines for subjects with hyperuricemia in order to prevent PEW. Establishing the existence of a phenomenon of reverse epidemiology with respect to UA levels in HD patients would require further studies with larger samples.

Limitations: We did not evaluate dietary supplements and drugs consumption except for allopurinol so we cannot analyze the effect of drugs (such as diuretics, angiotensin-converting enzyme inhibitors, statins and fibrates) on UA; however, we did a sub-analysis without including subjects with an allopurinol prescription and the results were similar. Furthermore, we did not register inflammatory markers such as white blood cells and C reactive protein. Finally, it is important to mention that all hemodialysis patients in this study had nutritional advice (AEC trained both dietitians); however, to our best knowledge, the same is not true for all hemodialysis units in our country and could be considered a bias factor in the study.

This research exposes how UA can be an indicator of the nutritional status associated with a greater antioxidant capacity. UA could be used as a marker of nutritional status, together with serum albumin, in environments where there are not enough nutritional resources to identify patients with nutritional risk in an inexpensive, easy and simple way.

## 5. Conclusions

In summary, patients with hyperuricemia had higher antioxidant capacity and less oxidative damage, and they also had better nutritional status in general, mainly according to impedance vectors. Furthermore, a positive association was found between hyperuricemia and albumin levels >3.5 g/dL. More studies are needed to stablish a cut point of UA levels in the renal population, similar to the URRAH project [13] in the hypertensive subject, and to confirm and clarify whether elevated uric acid could be an indicator of increased antioxidant capacity or better nutritional status in this population.

## Figures and Tables

**Table 1:** General characteristics in normo and hyperuricemic patients on hemodialysis.

	All Patients (*n* = 93)	Normouricemic Patients (*n* = 26)	Hyperuricemic Patients (*n* = 67)	*p*
Age (years)	40 (29–52)	47 (31–61)	39 (29–47)	0.026
Sex (M%)	50 (54)	18 (69.2)	32 (47.8)	0.062
*Comorbidities*				
Diabetes mellitus *n (%)*	24 (25.8)	8 (30.8)	16 (23.9)	0.496
Hypertension *n (%)*	57 (61.3)	18 (69.2)	39 (58.2)	0.355
*Allopurinol Consumption*				
Yes *n (%)*	8 (8.6)	1 (3.8)	7 (10.4)	0.308
Dialysis session time (min)	215.3 ± 17.3	211.7 ± 15.5	216.7 ± 17.9	0.195
Ultra-filtered (mL)	2000 (1353–2500)	2350 (1950–2564)	2000 (1200–2300)	0.051
Dialysis vintage (months)	39 (21–79)	47 (24–85)	38 (20–72)	0.300
*Sessions/Week (days)*				
3 *n (%)*	85 (91.4)	25 (96.2)	61 (91)	0.663
2 *n (%)*	7 (7.5)	1 (3.8)	5 (7.5)	
1 *n (%)*	1 (1.1)	0	1 (1.5)	
*Vascular Access*				
AF *n (%)*	57 (61.3)	16 (61.5)	41 (61.2)	1.0
Catheter *n (%)*	36 (38.7)	10 (38.5)	26 (38.8)	1.0
Right arm *n (%)*	51 (54.8)	14 (53.8)	37 (55.2)	1.0
*Filter*				
F18 *n (%)*	8 (8.6)	0	8 (11.9)	0.092
F80 *n (%)*	69 (74.2)	23 (88.5)	46 (68.7)	
F180 *n (%)*	16 (17.2)	3 (11.5)	13 (19.4)	
*Dialysis Efficacy*				
Kt/V	1.46 (1.25–1.77)	1.43 (1.18–1.67)	1.46 (1.25–1.79)	0.436
URR *(%)*	71 (65–79)	70 (65–74)	72 (66–81)	0.126

ESRD—end stage renal disease; AF—arteriovenous fistula; F18—filter number 18; F80—filter number 80; F180—filter number 180; URR —urea reduction rate; min—minutes. Continuous variables are expressed as mean ± SD or median with interquartile range in case of non-normally distributed data, and categorical variables are expressed as a percentage. *p* < 0.05 was considered statistically significant.

**Table 2:** Plasma antioxidant capacity and oxidant damage in normo and hyperuricemic patients on hemodialysis.

	Normouricemic Patients (*n* = 26)	Hyperuricemic Patients (*n* = 67)	*p*
DPPH^●^ scavenging (mM TE)	1.0495 ± 0.1226	1.1385 ± 0.1681	0.007
ORAC (μM TE)	2337.8 ± 370.7	2441.7 ± 313.7	0.213
MDA (nmol/mL)	12.7 ± 4.6	0.6 ± 13.5	0.020

DPPH^●^—2,2-diphenyl-1-picrylhidrazyl radical; ORAC—oxygen radical absorbance capacity; MDA—malondialdehyde; TE—Trolox equivalents. DPPH^●^ scavenging and ORAC assays were made by duplicate and MDA determination was made once. Results are expressed as mean ± SD. *p* < 0.05 was considered statistically significant.

**Table 3:** Biochemical and nutritional parameters in normo and hyperuricemic patients on hemodialysis.

	Normouricemic Patients (*n* = 26)	Hyperuricemic Patients (*n* = 67)	*p*
***Biochemical***			
Creatinine (mg/dL)	10.5 (8.1–11.8)	11.9 (9.6–14.5)	0.004
Albumin (g/dL)	3.8 ± 0.3	3.78 ± 0.4	0.771
BUN (mg/dL)	53 (41–60)	70 (54–90)	<0.001
Potassium (mg/dL)	5.0 ± 0.9	5.5 ± 0.7	0.014
Phosphorus (mg/dL)	5.0 (3.9–6.4)	5.1 (4.0–6.6)	0.942
Glucose (mg/dL)	85 (72–109)	83 (75.5–103)	0.834
Hemoglobin (mg/dL)	9.3 ± 2.01	9.9 ± 2.3	0.218
Hematocrit (%)	30.6 (25–34)	31.8 (26–35.6)	0.475
Transferrin (mg/dL)	213.4 ± 45.7	196.7 ± 54.1	0.148
***Nutritional scores***			
MIS score	4.5 (3–6)	5 (3–6)	0.785
Normal nutrition state/Mild malnutrition *n (%)*	17 (65.4)	47 (70.1)	0.803
Moderate malnutrition *n (%)*	7 (26.9)	17 (25.4)	
Severe malnutrition *n (%)*	2 (7.7)	3 (4.5)	
PEW (MIS) *n (%)*	9 (34.6)	24 (35.8)	0.656
PEW (ISRNM) *n (%)*	9 (34.6)	17 (25.4)	0.373
***Muscular functionality***			
Handgrip strength (kg)	25.3 ± 5.9	26.7 ± 8.5	0.366

BUN—blood urea nitrogen; MIS—malnutrition inflammation score; PEW—protein energy wasting; ISRNM—International Society of Renal Nutrition and Metabolism criteria. Continuous variables are expressed as mean ± SD or median with interquartile range in case of non-normally distributed data, and categorical variables are expressed as a percentage. *p* < 0.05 was considered statistically significant.

**Table 4:** Body composition and Dietary intake in normo and hyperuricemic patients on hemodialysis.

	Normouricemic Patients (*n* = 26)	Hyperuricemic Patients (*n* = 67)	*p*
***Anthropometrics***			
Dry weight (kg)	62.2 (56.4–70.9)	61.5 (53.9–71.6)	0.784
Height (m)	1.60 ± 0.05	1.59 ± 0.09	0.451
BMI (kg/m^2^)	25 (21.3–27.4)	24 (21.3–28.2)	0.983
AC (cm)	27.7 (25.7–29.6)	27.4 (25.6–31.5)	0.625
TSF (mm)	11.5 (10–17)	14 (11–18)	0.192
FM *(%)*	27.2 (20.5–31.6)	28.3 (20.9–32.9)	0.602
FFM *(%)*	72.8 (68.4–79.5)	71.7 (67.1–79.2)	0.602
***BIA***			
R/H (Ω/m)	323 (300.6–380)	360.4 (317.6–412.2)	0.139
Xc/H (Ω/m)	30.2 ± 10.4	37.6 ± 10.9	0.004
PA (°)	4.9 ± 1.2	5.8 ± 1.4	0.005
Cachexia diagnosis *n (%)*	14 (53.8)	19 (28.4)	0.021
***Dietetic***			
Energy (kcals/d)	1746.5 (1544–2055)	1746 (1293–2389)	0.869
Energy (kcals/kgAW/d)	28.5 (22.4–39.1)	27.4 (20.5–43.9)	0.777
Proteins (g/d)	64.1 (43.7–82.5)	65.3 (51.6–88.9)	0.340
Animal protein (g/d)	32.1 (20.3–42.8)	37.1 (30.8–54.1)	0.017
Vegetable protein (g/d)	21.8 (19.6–28.5)	21.8 (17.4–31.4)	0.911
Proteins (g/kgAW/d)	1.08 (0.68–1.53)	1.1 (0.7–1.6)	0.436
nPNA	0.83 (0.75–1.01)	1.03 (0.85–1.19)	0.013
Fructose (mg/d)	23.5 (16.1–40.8)	21.3 (14.1–33.9)	0.340

BMI—body mass index; AC—arm circumference; TSF—triceps skinfold; FM—fat mass; FFM—fat-free mass; BIA—bioelectrical impedance analysis; R/H—resistance over height: Xc/H—reactance over height; PA—phase angle; kcals/d—kilocalories/day; kcals/AW/d—kilocalories/kilograms of actual weight/day; g/d—grams/day; g/kgAW/d—grams/kilograms of actual weight/day;; nPNA—normalized protein nitrogen appearance. Continuous variables are expressed as mean ± SD or median with interquartile range in case of non-normally distributed data, and categorical variables are expressed as a percentage. *p* < 0.05 was considered statistically significant.

**Table 5:** Association between uric acid and nutritional status for albumin ≥3.5 g/dL in hemodialysis patients.

	Exp (B)	B	*p*	CI 95%	
				Lower	Upper
Hyperuricemia	11.792	2.467	0.032	1.24	112.1
Cachexia diagnosis	6.892	1.930	0.096	0.71	66.77
Kcals/d	1.045	0.044	0.001	1.02	1.074
Kcals/kgIW/d	0.084	−2.476	0.001	0.02	0.39
Proteins (g/d)	0.258	−1.355	0.001	0.12	0.58
Proteins (g/kgIW/d)	34.14	74.4	0.001	17.7	59.04
Handgrip strength (kg)	1.275	0.243	0.020	1.04	1.56

Kcals/d—kilocalories/day; Kcals/kg of ideal weight/day; g/d—grams/day; g/kg of ideal weight/day. CI—confidence interval. Model adjusted by sex and URR (urea reduction rate). *p* < 0.05 was considered statistically significant.

**Table 6:** Odds ratio for normouricemic patients to present malnutrition.

	OR	*p*	CI 95%	
			Lower	Upper
Xc/H < 35	2.79	0.028	1.1	7.071
nPNA < 1.0	3.78	0.007	1.4	10.208
Cachexia (yes)	2.95	0.021	1.156	7.518
Cr < 10 mg/dL	2.33	0.074	0.910	5.982
K < 5 mg/dL	2.97	0.023	1.136	7.772
PA < 5.5°	3.38	0.012	1.309	8.749
PEW (MIS)	0.95	0.913	0.367	2.452
Albumin ≥ 3.5 g/dL	1.2	0.363	0.815	2.197

OR—odds ratio; CI—confidence interval; Xc/H—reactance/height; nPNA—normalized protein nitrogen appearance; Cr—creatinine; K—potassium; PA—phase angle; PEW (MIS) —protein energy wasting diagnosed by malnutrition inflammation score; *p* < 0.05 was considered statistically significant.
